# N‐acetylcysteine ameliorates hematuria‐associated tubulointerstitial injury in 5/6 nephrectomy mice

**DOI:** 10.14814/phy2.15767

**Published:** 2023-07-07

**Authors:** Ajay Medipally, Min Xiao, Anjali A. Satoskar, Laura Biederman, Alana Dasgupta, Iouri Ivanov, Galina Mikhalina, Brad Rovin, Sergey V. Brodsky

**Affiliations:** ^1^ Department of Pathology The Ohio State University Wexner Medical Center Columbus Ohio USA; ^2^ Department of Pathology Nationwide Children's Hospital Columbus Ohio USA; ^3^ Rochester Regional Health Nephrology Rochester New York USA; ^4^ Department of Medicine The Ohio State University Wexner Medical Center Columbus Ohio USA

**Keywords:** 5/6 nephrectomy, interstitial fibrosis, mouse strain, N‐acetylcysteine

## Abstract

Chronic kidney disease (CKD) is characterized by increased interstitial fibrosis and tubular atrophy (IFTA) in the kidney. Chronic hematuria is a hallmark of several human kidney diseases and often is seen in patients on anticoagulation therapy. We had previously demonstrated that chronic hematuria associated with warfarin increases IFTA in 5/6 nephrectomy (5/6NE) rats, and such treatment increases reactive oxygen species (ROS) in the kidney. The goal of this study was to evaluate the effects of the antioxidant N‐acetylcysteine (NAC) on the progression of IFTA in 5/6NE mice. 5/6NE C57BL/6 and 5/6NE 129S1/SvImJ mice were treated with warfarin alone or with warfarin and NAC for 23 weeks. Serum creatinine (SCr), hematuria, blood pressure (BP), and ROSs in the kidney were measured; kidney morphology was evaluated. Warfarin doses were titrated to achieve prothrombin time (PT) increase to the levels seen with therapeutic human doses. Warfarin treatment resulted in an increased SCr, systolic BP, hematuria, expression of TGF‐ß and ROS in the kidney in both mouse strains. Tumor necrosis factor alpha (TNF‐ɑ) levels in the serum were increased in 5/6NE mice treated with warfarin. IFTA was increased as compared with control 5/6NE mice, and this increase in IFTA was more prominent in 129S1/SvImJ than in C57BL/6 mice. NAC ameliorated the warfarin‐associated increase in SCr and BP but not hematuria. IFTA, TGF‐ß, and ROS in the kidney as well as TNF‐ɑ levels in the serum were reduced in mice treated with NAC and warfarin as compared to mice treated with warfarin alone. NAC mitigates the increase in SCr and IFTA in mice with chronic hematuria by reducing oxidative stress in the kidney. This data open novel possibilities for treatments in CKD patients.

## 1 INTRODUCTION

Chronic kidney disease (CKD) is a common outcome of different kidney diseases. More than 37 million adults in the United States have CKD, (Centers for Disease C, and Prevention, 2007; Shahinian et al., 2013) and CKD is one of the leading cause of death in North America (Lozano et al., 2013). Unfortunately, there is no effective treatment for CKD; renal replacement therapy (dialysis) or transplantation are the only currently available options. Therefore, prevention of the CKD progression is one of the important tasks that clinicians face. CKD is the result of glomerular, vascular, and tubulointerstitial diseases. All of these conditions lead to increased interstitial fibrosis and tubular atrophy (IFTA). Several kidney diseases are characterized by hematuria and exposure of the tubular epithelial cells to free hemoglobin. We recently demonstrated that chronic hematuria increases the progression of interstitial fibrosis in 5/6 nephrectomy (5/6NE) rats, and the mechanisms of such progression are associated with the epithelial mesenchymal transition of the tubular epithelial cells (Xiao et al., 2021). Red blood cells (RBC) may release free hemoglobin, which may affect tubular epithelial cells by an increased oxidative stress (Patel et al., 1996). Oxidative stress has also been shown to play an important role of in the pathogenesis of acute kidney injury (AKI) associated with glomerular hemorrhage (Ware et al., 2013). Murine populations provide better models than rats to study the pathogenesis of different diseases since genetic manipulations in rats are technically challenging. Several previous reports demonstrated that 5/6NE 129S1/SvImJ mice are more susceptible to the development of CKD than 5/6NE C57BL/6 mice (Hamzaoui et al., 2020; Ma & Fogo, 2003).

The aim of the current study was to investigate the role of oxidative stress in the CKD progression in 5/6NE murine models.

## 2 MATERIALS AND METHODS

These studies have been approved by the Institutional Animal Care and Use Committees (IACUC) at the Ohio State University.

### 2.1 Experimental model

129S1/SvImJ mice were obtained from the Jackson laboratory, and C57BL/6 mice were obtained from the Charles River Laboratories. A 5/6NE procedure was performed in 3–4 months old (20–25 g) male mice as we described previously (Medipally et al., 2020). Briefly, animals were anesthetized with isoflurane/oxygen (1:5), and, through a middle laparotomy, a right nephrectomy was performed followed by resection of two thirds of the left kidney. The wound was closed with 4–0 proline, and the animals were kept on the standard rodent diet with free access to water.

Warfarin (Millipore Sigma; catalog# PHR1435) was given per os in drinking water in the dose of 0.5 mg/kg/day (for 129S1/SvImJ mice) and 1.0 mg/kg/day (for C57BL/6 mice) starting 3 weeks after 5/6 nephrectomy to allow recovery from the surgery and wound healing in the remnant kidney. N‐acetylcysteine (NAC) (Millipore Sigma; Catalog # A7250) was given per os in the dose of 30 mg/kg/day. The animal weight and the water consumption per each mouse were measured daily to calculate the dosage of the drugs. The warfarin dose was selected based on our previous data (Medipally et al., 2020) and pilot studies to increase PT between 1.5 and 3 times, mimicking PT goals in human therapeutic protocols. The control group included 5/6NE mice that were kept on water. Animals were monitored for 23 weeks after the ablative surgery.

### 2.2 Biochemical and hematological analysis

Blood (from the submandibular vein, 100 μL/time, using a 23‐gauge needle) and urine (spontaneous urine) samples were collected weekly and analyzed at the same day. Hematuria was measured by Siemens Multistix 5 (Siemens Healthcare Diagnostics Inc.) and expressed in a semiquantitative scale from 0 to 3, where score 0 is absent, 1+ is trace, 2+ is moderate, and 3+ is large. The animals were sacrificed at week 23 after 5/6NE, and the remnant kidney was dissected for histology and other studies. Histology of the kidney was evaluated on 2–3 mcm sections of paraffin‐embedded tissue stained with hematoxylin and eosin (H&E) and trichrome stains. The scarred areas of the kidney related to the surgical procedure were excluded from the analysis.

Serum creatinine was measured based on the Jaffe reaction using a creatinine reagent assay kit (Raichem) according to the manufacturer protocol. Briefly, 10 μL of serum was mixed with 200 μL of working reagent at 37°C in a 96‐well plate, and the absorbance was read at 510 nm at 60 sec and 120 sec on a microplate reader (Molecular Devices).

Prothrombin time (PT) was measured using the Biobsae coagulation analyzer (model COA01; Genprice Inc.) based on the manufacturer protocol as we reported previously (Medipally et al., 2020; Ozcan et al., 2012). In brief, blood was collected to a tube containing 3.8% sodium citrate as the anticoagulant with a ratio of 9:1. The blood was centrifuged at 1850 RCF for 15 min, 0.05 mL of plasma was placed in the incubation station for 2 min, 0.1 mL of warm thromboplastin was added, and the clotting time was recorded.

A “surrogate” INR (sINR) was used, by comparing PT before and after the treatment, as described previously (Medipally et al., 2020; Ozcan et al., 2012; Ware et al., 2011). The average PT in 25 mice prior to 5/6NE for each strain was used as the normal PT time.

TNF‐ɑ levels in the blood were measured by using Invitrogen (ThermoFisher Scientific; catalog # BMS607‐3) TNF alpha Mouse ELISA Kit following the manusfacturer protocol. Briefly, 50 μL of serum obtained from the mice at the end of the study were applied to a well of the microplate. After incubation for 30 min at room temperature, reaction was stopped and the absorbance at 450 nm was read by using a microplate reader (Versa Max, Molecular Devices).

### 2.3 Reactive oxygen species (ROS) determination

ROS were analyzed in the renal cortex by using the Protein Carbonyl Assay kit (Cayman Chemical Company) (Levine et al., 1994) based on the manufacturer protocol. To briefly summarize, 250 mg of renal cortex was homogenized in ice‐cold 2‐(N ‐morpholino)ethanesulfonic acid (MES) buffer (pH 6.7) and centrifuged at 10,000×g for 15 min at 4°C. Then 0.8 mL of 2,4‐dinitrophenylhydrazine (DNPH) was added to the supernatant. For control samples 0.8 mL of 2.5 M HCL was added. All samples were incubated in the dark at room temperature for 1 h. The samples were precipitated first with 1 mL of 20% trichloroacetic acid (TCA) followed by 10% TCA and centrifuged at 10,000 g for 10 min. The pellet was washed thrice with 1 mL of ethanol‐ethyl acetate (1:1;v/v) to remove free DNPH reagent and centrifuged for 10 min at 10,000 g. The protein pellet was re‐suspended in 0.5 mL of guanidine hydrochloride with vortexing. Samples were then centrifuged at 10,000×g for 10 min at 4°C. The concentration of DNPH in the supernatant was determined spectrophotometrically at 370 nm (Versa Max, Molecular Devices), and the molar absorption coefficient of 22,000 M^−1^ cm^−1^ was used to quantify the levels of protein carbonyls. Protein carbonyl concentration was determined in the samples by the equation: Protein Carbonyl (nmol/mL) = (CA)/(*0.011 μM‐1)(500/200 μL), where CA is the corrected absorbance of the samples. Protein carbonyl concentration was calculated per gram of wet tissue.

### 2.4 Immunohistochemistry

Transforming growth factor beta (TGF‐ß) expression in the kidney was analyzed by immunohistochemistry on paraffin embedded tissue after antigen retrieval, according to the manufacturer protocol. Briefly, tissue was deparaffinized and antigen retrieval was performed by microwaving the slides for 10 min in Sodium Citrate buffer (pH 6.0). To block endogenous peroxidase activity, the tissue sections were incubated in 3.0% hydrogen peroxide in methanol for 15 min. Permeabilization was performed by incubating tissue with permeabilization buffer containing 1% animal serum and 0.4% Triton X‐100 in PBS for 10 min. Tissue was incubated with primary anti‐ TGF‐ß antibody (Novus Biologicals; catalog # NBP1‐80289) at the concentration 10 μg/mL overnight at 4oC followed by incubation for 1 h at room temperature with secondary antibody (Santa Cruz Biotechnology; cat # sc‐2313), dilution 1:1000. Stained slides were analyzed and the expression of TGF‐ß was calculated as the percentage in the tubular epithelial cells.

### 2.5 Blood pressure measurement

Blood pressure was measured by a tail cuff method using a blood pressure monitor (IITC Life Sciences Inc.), as we reported earlier (Ware et al., 2015). The systolic and diastolic BP were determined using the Blood Pressure Data Acquisition Software (IITC Life Sciences Inc. Version 1.35).

### 2.6 Statistical analysis

Results are presented as mean ± standard deviation (SD) if not otherwise specified. Differences between two groups were analyzed by the two‐paired Student *t*‐test or two‐way ANOVA test, where applicable.

## 3 RESULTS

### 3.1 Warfarin treatment and kidney function in 5/6NE mice

**FIGURE 1:**
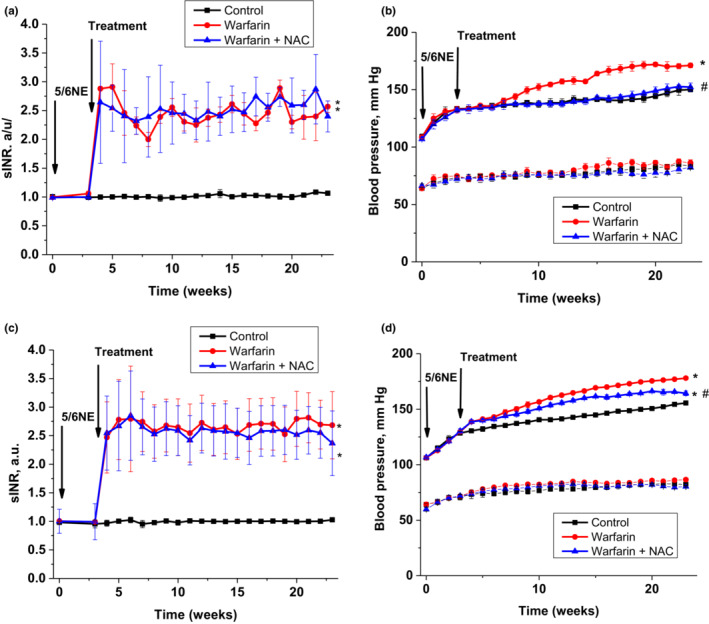
Changes in prothrombin time and blood pressure in mice treated with warfarin and N‐acetylcysteine (NAC). (a) Changes in prothrombin time, calculated as sINR, in 5/6 nephrectomy (5/6NE) C57BL/6 mice treated per os with warfarin 1.0 mg/kg/day alone (circle, *n* = 7) or with warfarin 1.0 mg/kg/day and NAC 30 mg/kg/day (triangle, *n* = 7). Control (square, *n* = 6) are vehicle‐treated 5/6NE C57BL/6 mice. (b) Changes in systolic blood pressure (upper 3 lines) and diastolic blood pressure (lower 3 lines) in 5/6 nephrectomy (5/6NE) C57BL/6 mice treated per os with warfarin 1.0 mg/kg/day alone (circle, *n* = 7) or with warfarin 1.0 mg/kg/day and NAC 30 mg/kg/day (triangle, *n* = 7). Control (square, *n* = 6) are vehicle‐treated 5/6NE C57BL/6 mice. (c) Changes in prothrombin time, calculated as sINR, in 5/6NE 129S1/SvImJ mice treated per os with warfarin 0.5 mg/kg/day alone (circle, *n* = 7) or with warfarin 0.5 mg/kg/day and NAC 30 mg/kg/day (triangle, *n* = 7). Control (square, *n* = 6) are vehicle‐treated 5/6NE 129S1/SvImJ mice. (d) Changes in systolic blood pressure (upper 3 lines) and diastolic blood pressure (lower 3 lines) in 5/6NE 129S1/SvImJ mice treated per os with warfarin 0.5 mg/kg/day alone (circle, *n* = 7) or with warfarin 0.5 mg/kg/day and NAC 30 mg/kg/day (triangle, *n* = 7). Control (square, *n* = 6) are vehicle‐treated 5/6NE 129S1/SvImJ mice. *p < 0.005 as compared to control mice, ^#^ *p* < 0.05 as compared to warfarin‐only treated mice.

Animals were treated with warfarin starting 3 weeks after the ablative surgery for a total of 20 weeks (*n* = 7 in each mouse strain in warfarin and warfarin + NAC groups, *n* = 6 in control group). Warfarin treatment resulted in a PT increase in 5/6NE mice in both strains. sINR was increased between 2 and 3 times from baseline, mimicking therapeutic protocols in humans. NAC did not affect warfarin—induced changes in sINR (Figure 1a,c). Consistent with our previous findings, systolic blood pressure was increased in 5/6NE mice in both strains treated with warfarin, but diastolic blood pressure remained stable after the warfarin treatment (Ware et al., 2015). The warfarin associated systolic blood pressure increase was prevented by NAC administration and this affect was more pronounced in 5/6NE C57BL/6 mice than in 5/6NE 129S1/SvImJ mice (Figure 1b,d).

**FIGURE 2:**
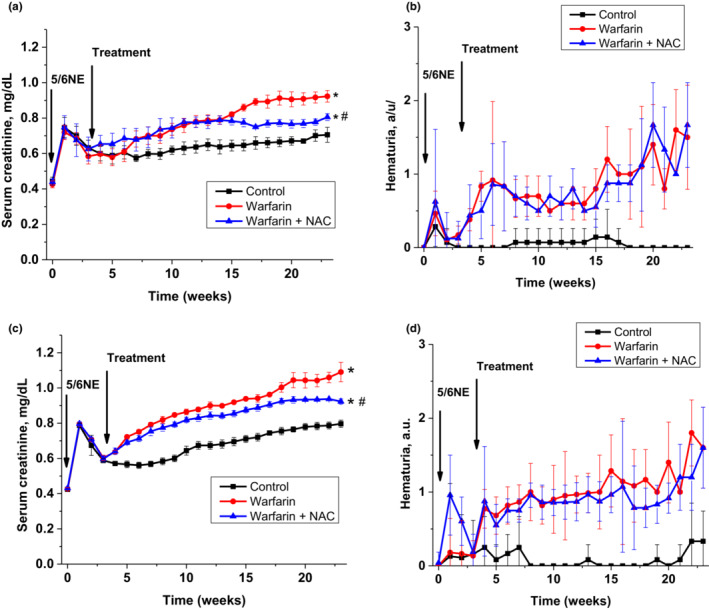
Changes in serum creatinine and hematuria in mice treated with warfarin and N‐acetylcysteine (NAC). (a) Changes in serum creatinine in 5/6 nephrectomy (5/6NE) C57BL/6 mice treated per os with warfarin 1.0 mg/kg/day alone (circle, *n* = 7) or with warfarin 1.0 mg/kg/day and NAC 30 mg/kg/day (triangle, *n* = 7). Control (square, *n* = 6) are vehicle‐treated 5/6NE C57BL/6 mice. (b) Changes in hematuria in 5/6 nephrectomy (5/6NE) C57BL/6 mice treated per os with warfarin 1.0 mg/kg/day alone (circle, *n* = 7) or with warfarin 1.0 mg/kg/day and NAC 30 mg/kg/day (triangle, *n* = 7). Control (square, *n* = 6) are vehicle‐treated 5/6NE C57BL/6 mice. (c) Changes in serum creatinine in 5/6NE 129S1/SvImJ mice treated per os with warfarin 0.5 mg/kg/day alone (circle, *n* = 7) or with warfarin 0.5 mg/kg/day and NAC 30 mg/kg/day (triangle, *n* = 7). Control (square, *n* = 6) are vehicle‐treated 5/6NE 129S1/SvImJ mice. (d) Changes in hematuria in 5/6NE 129S1/SvImJ mice treated per os with warfarin 0.5 mg/kg/day alone (circle, *n* = 7) or with warfarin 0.5 mg/kg/day and NAC 30 mg/kg/day (triangle, *n* = 7). Control (square, *n* = 6) are vehicle‐treated 5/6NE 129S1/SvImJ mice. Hematuria was measured by Siemens Multistix 5 (Siemens Healthcare Diagnostics Inc, Tarrytown, NY) and expressed in a semiquantitative scale from 0 to 3, where score 0 is absent, 1+ is trace, 2+ is moderate and 3+ is large. *p < 0.005 as compared to control mice, ^#^ *p* < 0.05 as compared to warfarin‐only treated mice.

Serum creatinine gradually increased in the post‐operative interval in both mice strains. Increase in serum creatinine in 5/6NE 129S1/SvImJ mice was higher than in 5/6NE C57BL/6 mice at 23 weeks after the ablative surgery (0.8 ± 0.01 mg/dL vs. 0.7 ± 0.01 mg/dL, accordingly, *p* = 0.0013). Warfarin treatment resulted in a significant acceleration of serum creatinine rise in both mice strains (*p* < 0.01, Figure 1a,c). By the end of 23 weeks, serum creatinine was 1.1 ± 0.02 mg/dL in 5/6NE 129S1/SvImJ mice treated with warfarin and 0.8 ± 0.01 mg/dL in 5/6NE C57BL/6 mice treated with warfarin (*p* = 0.003). Treatment with NAC ameliorated the warfarin‐associated increase in serum creatinine in 5/6NE in both mouse strains (Figure 2a,c).

Control 5/6NE C57BL/6 mice did not have hematuria, whereas mild hematuria was seen in control 5/6NE 129S1/SvImJ mice after 20 weeks post‐surgery (Figure 2b,d). Hematuria was increased in 5/6NE mice in both strains treated with warfarin and reached mild‐to‐moderate levels by the end of the studies. NAC did not affect warfarin‐associated hematuria in either 5/6NE mouse strain (Figure 2b,d).

### 3.2 Histologic findings in 5/6 nephrectomy mice treated with warfarin and N‐acetylcysteine

**FIGURE 3:**
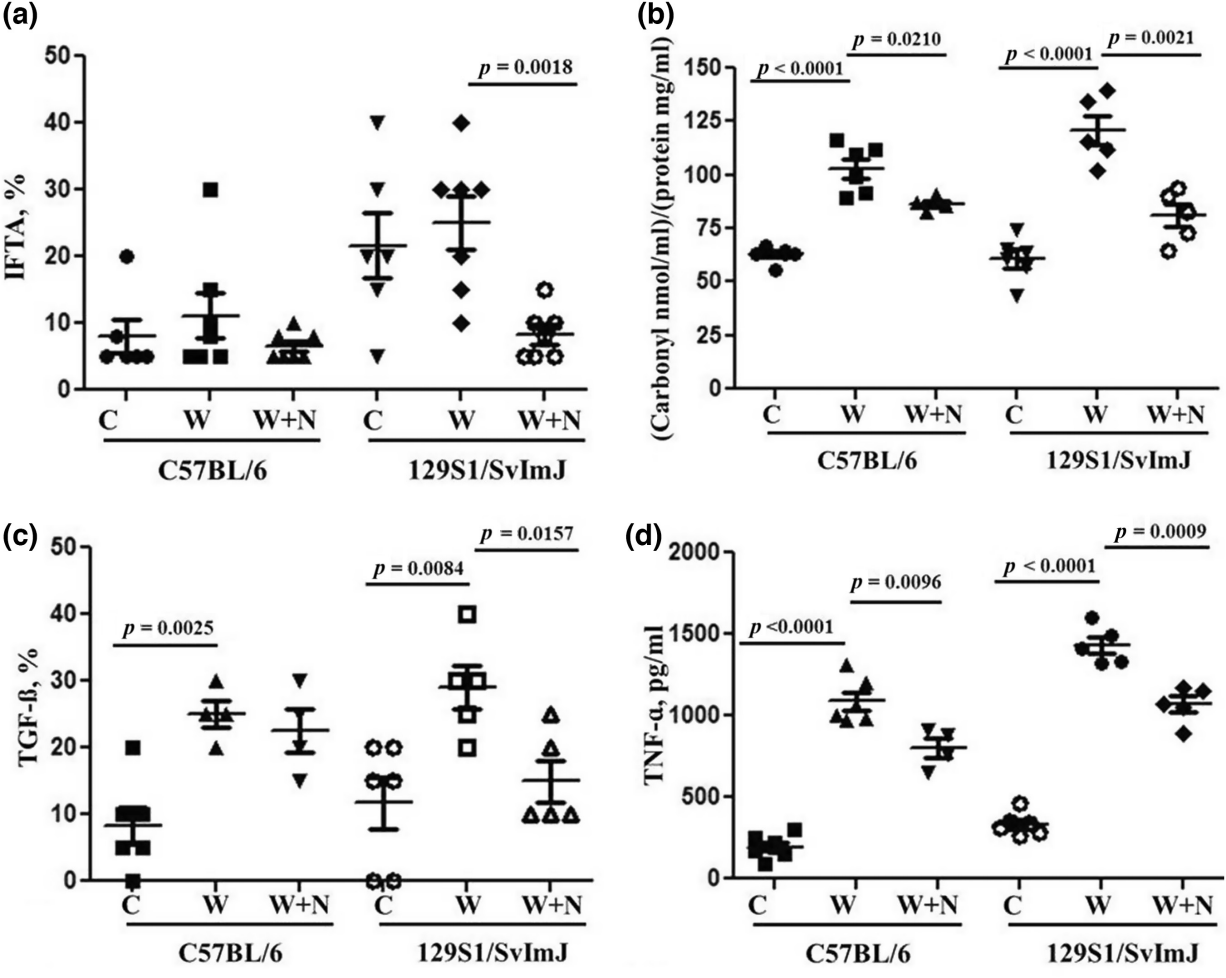
Changes in interstitial fibrosis and tubular atrophy (IFTA), reactive oxygen species, tissue expression of TGF‐ß and serum levels of TNF‐ in mice treated with warfarin and N‐acetylcysteine (NAC). (a) Changes in IFTA (calculated as the percentage of the renal cortex) in 5/6NE C57BL/6 and 5/6NE 129S1/SvImJ mice treated per os with warfarin alone or with warfarin and NAC. (b) Reactive oxygen species were measured as protein carbonyl concentration in the renal cortex in in 5/6NE C57BL/6 and 5/6NE 129S1/SvImJ mice treated per os with warfarin alone or with warfarin and NAC. (c) Changes in the expression of TGF‐ß in the kidney in 5/6NE mice treated with warfarin alone and warfarin + NAC. Percentage of the expression in the tubular epithelial cells was evaluaterd. (d) Serum TNF‐ɑ levels in 5/6NE mice treated with warfarin and warfarin + NAC. Blood was collected at the end of the studies (23 weeks after the ablative surgery).

**FIGURE 4:**
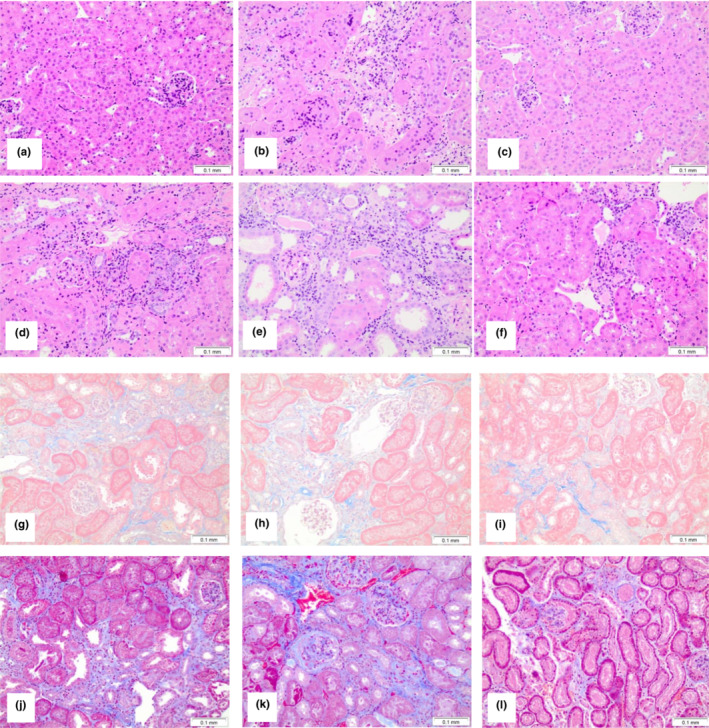
Histologic changes in mice treated with warfarin and N‐acetylcysteine (NAC). Representative images from the kidneys obtained from mice at 23 weeks after the ablative surgery and treated with warfarin or warfarin and NAC. (a, g) Control 5/6NE C57BL/6 mice; (b, h) 5/6NE C57BL/6 mice treated with warfarin 1.0 mg/kg/day; (c, i) 5/6NE C57BL/6 mice treated with warfarin 1.0 mg/kg/day and NAC 30 mg/kg/day. (d, j) control 5/6NE 129S1/SvImJ mice; (e, k) 5/6NE 129S1/SvImJ mice treated with warfarin 0.5 mg/kg/day; (f, l) 5/6NE 129S1/SvImJ mice treated with warfarin 0.5 mg/kg/day and NAC 30 mg/kg/day. (a–f) Hematoxylin and Eosin stain, (d–l) Masson's trichrome stain. Magnification 200×.

All 5/6NE mice developed IFTA at 23 weeks after the surgery. IFTA was more prominent in control 5/6NE 129S1/SvImJ mice as compared to control 5/6NE C57BL/6 mice (20.0 ± 6.5% as compared to 9.7 ± 2.7%, respectively, though this difference did not reach statistical significancy, *p* = 0.1330). Warfarin treatment slightly increased IFTA in both mouse strains (11.0 ± 10.8% vs. control 9.7 ± 2.7%, *p* = 0.8079 in 5/6NE C57BL/6 mice and 25.0 ± 12.3% vs. control 20.0 ± 6.5%, *p* = 0.5733 in 5/6NE 129S1/SvImJ mice) (Figures 3a and 4). In 5/6NE C57BL/6 mice treated with warfarin and NAC, IFTA was 6.3 ± 2.5% as compared to 11.0 ± 10.8% in warfarin only treated mice (*p* = 0.4248) and 9.7 ± 2.7% in control 5/6NE C57BL/6 mice (*p* = 0.3797). Treatment with NAC not only prevented the warfarin‐induced increase in IFTA in both mouse strains, but IFTA in 5/6NE mice treated with warfarin and NAC was lower than in control 5/6NE mice, though it was not statistically significant. Additionally, in 5/6NE 129S1/SvImJ mice the beneficial effect of NAC on the IFTA progression was more pronounced; IFTA was 8.3 ± 4.1% in 5/6NE 129S1/SvImJ mice treated with both warfarin and NAC as compared to 25.0 ± 12.3% in mice treated with warfarin alone (*p* = 0.0016) and 20.0 ± 6.5% in control 5/6NE 129S1/SvImJ mice (*p* = 0.0910) (Figures 3a and 4).

Reactive oxygen species (ROS) were measured in the cortex in the kidneys obtained from both mouse strains. The right kidney removed during the 5/6NE was served as baseline. There was no increase in ROS in control 5/6NE mice in both strains by 23 weeks after the ablative surgery. Treatment with warfarin significantly increased protein carbonyl concentration in the kidney in both mouse strains. In 5/6NE C57BL/6 mice treated with warfarin, protein carbonyl concentration in the kidney were 103.0 ± 11.1 nmol/mg as compared to 63.0 ± 3.6 nmol/mg in control 5/6NE C57BL/6 mice (*p* < 0.0001). In 5/6NE 129S1/SvImJ mice treated with warfarin, protein carbonyl concentration in the kidney was 120.8 ± 15.8 nmol/mg as compared to 60.8 ± 10.4 nmol/mg in control 5/6NE 129S1/SvImJ mice (*p* < 0.0001) (Figure 3b). Treatment with NAC reduced but did not abolish warfarin‐associated increase in protein carbonyl concentration in the kidney. In 5/6NE C57BL/6 mice treated with warfarin and NAC, protein carbonyl concentration in the kidney were 86.3 ± 3.4 nmol/mg as compared to 103.0 ± 11.1 nmol/mg in 5/6NE C57BL/6 mice treated with warfarin only (*p* = 0.021). In 5/6NE 129S1/SvImJ mice treated with warfarin and NAC, protein carbonyl concentration in the kidney were 81.07 ± 12.2 nmol/mg as compared to 120.8 ± 15.8 nmol/mg in 5/6NE 129S1/SvImJ mice treated with warfarin only (*p* = 0.0021) (Figure 3b).

TGF‐ß expression in the kidney increased in 5/6NE mice in both mouse strains after treatment with warfarin. Treatment with NAC reduced TGF‐ß expression in the kidney in 5/6NE mice treated with warfarin, which was more prominent in 129S1/SvImJ mice (Figure 3c).

Treatment with warfarin increased serum levels of inflammatory biomarker TNF‐ɑ in 5/6NE mice in both mouse strains. NAC decreased this warfarin‐induced increase in TNF‐ɑ serum levels in both 5/6NE C57BL/6 and 129S1/SvImJ mice (Figure 3d).

## 4 DISCUSSION

To the best of our knowledge, this is the first study describing the progression of interstitial fibrosis and beneficial effects of NAC in mice treated with an anticoagulant. We used two different mouse strains that are widely used in research. Both C57BL/6 and 129S1/SvImJ mice are wild‐type mice for many knockout mice. Therefore, our data would be useful for studying the mechanisms of interstitial fibrosis in the kidney by using knockout mice developed from these background strains.

IFTA is the common outcome of many kidney diseases. Previously we demonstrated that chronic hematuria in 5/6NE rats treated with warfarin increases IFTA and is associated with an increased oxidative stress and epithelial mesenchymal transition in the kidney (Xiao et al., 2021). In the current study we used C57BL/6 and 129S1/SvImJ mouse strains because these mice have different progression of IFTA in response to 5/6NE (Hamzaoui et al., 2020; Ma & Fogo, 2003). 5/6NE C57BL/6 had only mild IFTA 24 weeks after the ablative surgery, but 5/6NE 129S1/SvImJ mice had marked IFTA at the same time point after the ablative surgery (Ma & Fogo, 2003). In our studies, we obtained similar results; IFTA was only slightly increased in 5/6NE C57BL/6 23 weeks after the ablative surgery, but a more prominent increase was seen in 5/6NE 129S1/SvImJ mice (Figures 3a and 4). Treatment with warfarin resulted in an increase in hematuria, more prominent IFTA, and more prominent increase in serum creatinine in both mouse strains after 5/6NE as compared to 5/6NE control mice.

Our studies show beneficial effects of NAC on serum creatinine levels and the progression of IFTA in 5/6NE mice in both strains. In 5/6NE C57BL/6 23 and 5/6NE 129S1/SvImJ mice, treatment with NAC ameliorated warfarin‐associated increases in serum creatinine (Figure 2). In both mouse strains, IFTA was lower in 5/6NE mice after treatment with warfarin and NAC as compared to 5/6NE animals treated with warfarin alone, though only in 5/6NE 129S1/SvImJ mice was it statistically significant (Figures 3a and 4). This was due to reduction in oxidative stress measured as reactive oxygen species (protein carbonyl concentration) in the kidney. Indeed, warfarin increased renal protein carbonyl concentration in 5/6NE mice, and treatment with NAC significantly reduced the warfarin‐associated protein carbonyl concentration increase in the kidney in both mouse strains (Figure 3b). Beneficial effects of NAC on the reduction of IFTA have been reported in other CKD models; the reduction of IFTA in mice (Honma et al., 2020) and rats (Shimizu et al., 2008) with ureteral obstruction were reported by several investigators. In other models of CKD such as cisplatin‐induced kidney injury in mice (Li et al., 2019), angiotensin II‐mediated renal fibrosis in mice (Shen et al., 2016), cadmium‐induced CKD in mice (Dong et al., 2023), streptozotocin‐induced diabetic nephropathy in rats (Nogueira et al., 2018), and diabetic nephropathy in fatty rats (Lee et al., 2016), IFTA was also reduced in animals treated with NAC. Interestingly, IFTA was decreased in transgenic mice with dilated cardiomyopathy that were treated with NAC as compared to wild‐type mice (Giam et al., 2017), suggesting that this treatment is effective not only in experimental models of direct kidney injury but also in other diseases associated with extrarenal etiologies of CKD. Several case reports show improving kidney function after treatment with NAC in patients with biopsy‐proven hemosiderosis in the kidney (Ackermann et al., 2004; Lee et al., 2020). In CKD patients, especially in those on chronic dialysis, the beneficial effects of treatments with different antioxidants on serum C‐reactive protein levels (a biomarker of kidney dysfunction) have been reported (Supriyadi et al., 2023). One of the possible mechanisms of NAC‐associated reduction in IFTA could be a decreased inflammatory response. Indeed, levels of TNF‐ɑ in the serum in mice treated with NAC and warfarin were lower as compared to the mice treated with warfarin alone (Figure 3d).

Warfarin affects vitamin K dependent proteins, including matrix Gla protein (MGP). MGP is a potent inhibitor of vascular calcification. Knockout MGP mice develop spontaneous arterial calcification at an early age (Nigwekar et al., 2017). Warfarin treatment in some patients is associated with calciphylaxis, a rare but frequently fatal vascular calcification disorder in patients receiving dialysis (Brandenburg et al., 2011). It has been shown that renal MGP expression is increased in human and experimental (5/6 nephrectomy in rats) CKD (Miyata et al., 2018). Interstitial expression of MGP correlated with interstitial fibrosis, tubular atrophy, acute tubular injury, and interstitial inflammation in 5/6 nephrectomy rats. In humans, higher levels of MGP expression in the interstitium in the kidney were associated with an increased risk for end‐stage kidney disease (Miyata et al., 2018). It is possible that MGP is responsible for mild increase in IFTA, as compared to control 5/6NE mice, in mice treated with warfarin and NAC (Figure 3a).

It has been shown that C57BL/6 mice are resistant to 5/6NE for up to 24‐weeks after the surgery (Ma & Fogo, 2003). We used C57BL/6 mice to evaluate the possibility that hematuria increases IFTA even in this fibrosis‐resistant strain. Our data indicate that in spite of similar hematuria and ROS in the kidneys as in 129S1/SvImJ mice, IFTA did not increase in C57BL/6 mice (Figures 2 and 3). Thus, C57BL/6 mice are resistant to oxidative stress and the chronic kidney injury in these mice develops by different mechanisms. These findings contribute to the pathogenesis of IFTA and further investigations are needed to investigate why C57BL/6 mice are resistant to IFTA development.

In addition to reduction of ROS in the kidney, there could be an additional mechanism for decreasing IFTA in our studies. We had earlier demonstrated that warfarin increases systolic blood pressure in 5/6NE rats (Ware et al., 2015). Similar increases in systolic blood pressure were observed in the current study in 5/6NE in both mouse strains treated with warfarin. Treatment with NAC prevented such warfarin‐induced increase in systolic blood pressure. It is well documented that hypertension is one of the main causes of CKD (Mallamaci, 2016; Sternlicht & Bakris, 2017; Weldegiorgis & Woodward, 2020). Thus, reduction of blood pressure in 5/6NE mice treated with warfarin could be partially responsible for decreased IFTA in these animals. Interestingly, there is evidence that increased oxidative stress in the kidney could increase peripheral blood pressure (Araujo & Wilcox, 2014), and the antioxidant action of NAC could be partially responsible for the reduction in systolic blood pressure in 5/6NE mice treated with warfarin.

One of the deficiencies of our work is that we used Jaffe colorimetric method to measure serum creatinine in mice. Even though this method is still in use in the clinical practice, it is well‐described by several different groups that this overestimates the creatinine in mice plasma as compared to HPLC or enzymatic assays (Dunn et al., 2004; Keppler et al., 2007). However, using the same read‐out method for all study groups we show dynamic changes of serum creatinine in mice and effects of different treatments. Therefore, if there is a systematic error associated with our method, then it is present in all the groups and the comparison between the groups show changes that are associated with treatments. Second limitation of our studies is that we did not have NAC‐only treated 5/6NE group. There are studies showing that NAC is protective in CKD including studies showing that NAC protects against 5/6 NE in rats (Shimizu et al., 2005). Thus, beneficial effect of NAC in warfarin‐treated mice could be due to the protective effect of NAC on 5/6 NE and independent of the hematuria‐related CKD. In our studies, we found that there was increase in ROS in the kidney in warfarin‐treated mice as compared to 5/6NE control mice that was prevented by NAC in both mouse strains (Figure 3b). IFTA in NAC + warfarin group was similar to that in control group, suggesting that NAC prevented warfarin‐induced increase in IFTA via reduction of warfarin‐induced oxidative stress in the kidney. It was accompanied by a decrease in inflammatory biomarker TNF‐ɑ levels in the serum in both mouse strains treated with NAC and warfarin as compared to warfarin treatment alone (Figure 3d), as well as decrease in the expression of TGF‐ß in the kidneys in 5/6NE mice treated with NAC and warfarin as compared to warfarin only treatment (Figure 3c).

In conclusion, we provide evidence that treatment with the antioxidant NAC decreases hematuria‐associated IFTA in the kidney in 5/6NE model of CKD in mice. Considering that many glomerular diseases are associated with chronic hematuria, this data lay the necessary groundwork for clinical trials to evaluate the benefits of antioxidative medications on the progression of CKD in IgA nephropathy, thin basement membrane nephropathy, and others.

## AUTHOR CONTRIBUTIONS

Ajay Medipally: conducted animal studies (surgeries), collected and analyzed samples, participated in data analysis, writing and reviewing the manuscript. Min Xiao: conducted animal studies, collected and analyzed samples, participated in data analysis, writing and reviewing the manuscript. Anjali A. Satoskar: participated in study design, data analysis, writing and reviewing the manuscript. Laura Biederman: participated in study design, data analysis, writing and reviewing the manuscript. Alana Dasgupta: participated in study design, data analysis, writing and reviewing the manuscript. Iouri Ivanov: participated in study design, data analysis, writing and reviewing the manuscript. Galina Mikhalina: participated in study design, data analysis, writing and reviewing the manuscript. Brad Rovin: participated in study design, data analysis, writing and reviewing the manuscript. Sergey V. Brodsky: oversees the entire study, designed experiments, performed data analysis, writing and reviewing the manuscript.

## ETHICS STATEMENT

The studies were approved by the Institutional Animal Care and Use Committees (IACUC) at the Ohio State University.
